# Review of Tolvaptan’s Pharmacokinetic and Pharmacodynamic Properties and Drug Interactions

**DOI:** 10.3390/jcm3041276

**Published:** 2014-11-12

**Authors:** Purav R. Bhatt, Elizabeth B. McNeely, Tess E. Lin, Kirkwood F. Adams, J. Herbert Patterson

**Affiliations:** 1 Division of Pharmacotherapy and Experimental Therapeutics (DPET), UNC Eshelman School of Pharmacy, University of North Carolina at Chapel Hill, CB# 7569 Rm 3212 Kerr Hall, Chapel Hill, NC 27599-7569, USA; E-Mails: purav243@gmail.com (P.R.B.); tesslin@unc.edu (T.E.L.); 2 Cardiovascular Team Lead Pharmacist, Department of Pharmacy, Rex Hospital, Raleigh, NC 27607, USA; E-Mail: elizabethannebeach@gmail.com; 3 Division of Cardiology, UNC Heart and Vascular, University of North Carolina at Chapel Hill, CB# 7569 Rm 3212 Kerr Hall, Chapel Hill, NC 27599-7569, USA; E-Mail: kfa@med.unc.edu

**Keywords:** tolvaptan, pharmacokinetic, pharmacodynamic, arginine vasopressin, aquaretic, CYP3A4, digoxin, heart failure, hyponatremia

## Abstract

Tolvaptan is an arginine vasopressin (AVP) antagonist that acts to increase excretion of free water (aquaresis) in patients without introducing electrolyte abnormalities or worsening renal function. It works via blockade of vasopressin-2 receptors at the renal collecting duct. Since the approval of tolvaptan for the treatment of hypervolemic and euvolemic hyponatremia in 2009, new studies have been reported to better characterize its pharmacokinetic and pharmacodynamic profile of tolvaptan. This paper is a review of both these clinical studies, as well as previous literature, in order to help guide appropriate clinical use of tolvaptan in patients. With appropriate monitoring of serum sodium, tolvaptan may be safely dose escalated from 15 mg once daily to a maximum effective dose of 60 mg once daily for multiple days, to achieve optimal aqauretic effects. In terms of drug interactions, co-administration of moderate to potent CYP3A4 inhibitors and inducers should be avoided. Tolvaptan should also be co-administered with caution and proper monitoring in the presence of *P*-glycoprotein substrate and strong inhibitors. Co-administration of tolvaptan with diuretic therapy did not appear to alter the aquaretic effect of tolvaptan; and was shown to be safe and well tolerated.

## 1. Introduction

The development of tolvaptan, an oral drug that is selective for vasopressin blockade at the V2-receptors in the renal collecting duct, has changed the management of hypervolemic and euvolemic hyponatremia. The increased use of tolvaptan has led to several studies assessing the changes in the pharmacokinetics (PK) and pharmacodynamics (PD) of tolvaptan when used with other classes of medications and different mode of administration. This article serves as a comprehensive review of the potential PK/PD alterations with the use of tolvaptan.

Tolvaptan (Samsca^®^, Otsuka America Pharmaceuticals, Rockville, MD, USA) was approved by the Food and Drug Administration (FDA) in 2009 for the treatment of clinically significant hypervolemic and euvolemic hyponatremia (serum sodium less than 125 mEq/L or less marked hyponatremia that is symptomatic and has resisted correction with fluid restriction), including patients with heart failure and syndrome of inappropriate antidiuretic hormone (SIADH) [1,2]. Tolvaptan has also been approved in 2014 for an additional indication by the Ministry of Health, Labor and Welfare of Japan for treatment of Autosmal Dominant Polycystic Kidney Disease (ADPKD), with this indication being under review in the United States and European Uion.

Euvolemic hyponatremia often is associated with SIADH [3], whereas hypervolemic hyponatremia commonly is associated with underlying conditions such as heart failure, cirrhosis, and renal failure [4,5,6,7,8]. Treatment of hyponatremia depends on the underlying cause of the sodium imbalance, volume status of the patient, acuteness of the onset of hyponatremia, and the presence and severity of symptoms [9]. Conventional treatments, such as fluid restriction, hypertonic saline, demeclocycline, and diuretics, have not been well tolerated when treating hyponatremia due to associated electrolyte abnormalities, neurohormonal activation, renal dysfunction, and possible increased mortality [9,10]. Because traditional treatments for hyponatremia are difficult for patients to tolerate, have marginal efficacy, and could result in electrolyte imbalances, vasopressin antagonists were developed specifically to counteract the abnormal pathophysiology resulting from an excess of arginine vasopressin (AVP) [11].

Arginine vasopressin (AVP), also known as antidiuretic hormone (ADH) or simply vasopressin, plays a critical role in the maintenance of arterial pressure and balance between sodium and water in the body. It is a neuropeptide synthesized in the hypothalamus and stored in the pituitary gland [12]. Usually there is very little secretion of AVP in low plasma osmolality states. However, secretion of AVP is stimulated in response to an increase in plasma osmolality or a decrease in arterial volume.

AVP acts at specific vasopressin receptors expressed in different tissues of the body to regulate homeostasis and maintain perfusion pressure. In the smooth muscle cells of arteries, AVP binds to the V1a receptor and causes smooth muscle contraction and arterial vasoconstriction, thereby maintaining perfusion pressure. In the renal tubular cells of the collecting duct, AVP binds to the V2 receptor and stimulates intracellular cyclic adenosine monophosphate (cAMP), which leads to activation of protein kinase A and synthesis and insertion of aquaporin-2 (AQP2) water channels in cells of the collecting duct. AQP2 channels allow reabsorption of water into the hypertonic medulla [13]. The action of AVP at the V2 receptor helps maintain plasma osmolality within the normal range by increasing free water reabsorption. In times of pathophysiologic stress, such as hyponatermia, AVP efficacy can be lowered by administering an AVP V2 receptor antagonist, which reduces the number of AQP2 channels inserted and thus increases the renal excretion of solute free water (aquaresis). Tolvaptan has been approved by the FDA to be initiated at a dose of 15 mg once daily. It can also be titrated by 15 mg every 24 hours to a maximum dosage of 60 mg once daily [2].

## 2. Tolvaptan Pharmacokinetics (PK)

At the time tolvaptan was approved by the FDA for the treatment of hypervolemic or euvolemic hyponatremia, data on tolvaptan’s PK and PD effects in humans were limited. The PK and PD profiles of tolvaptan after a single oral dose were reported by Shoaf *et al.* in two randomized, double-blind, placebo-controlled, ascending single-dose studies in primarily Caucasian healthy subjects [14]. Tolvaptan, at the tested doses of 60–480 mg, showed a dose-dependent increase in 72-hour cumulative urine output; however, cumulative urine output and aquaretic effect were similar for all doses tested within 12-hour post-dose [14]. In terms of safety, no dose-limiting toxicities were observed. Furthermore, serum aldosterone, plasma renin concentrations, and plasma AVP concentrations were not dose-dependently increased by tolvaptan, even at the highest doses studied. These data suggest that there is saturation in tolvaptan’s effect in urine output, and extended duration of effect with higher doses, which have been subsequently studied since tolvaptan’s FDA approval in 2009.

To investigate PK properties of tolvaptan at doses <60 mg, Kim *et al.* performed single- and multiple-dose studies, as well as a food-effect study, to further describe the relationship between tolvaptan PK and PD in healthy Japanese volunteers [15]. In the single-blind, ascending single-dose study, subjects were randomized to receive tolvaptan or placebo in fasted states at each sequential dose group. A total of 42 subjects received tolvaptan at doses of 15–120 mg, and 14 subjects received placebo. Safety assessments were reviewed at each dose level before dose escalation. Plasma and urine concentrations of tolvaptan were assessed using high-performance liquid chromatography-tandem mass spectrometry. Results showed that after a single dose of tolvaptan, maximum plasma tolvaptan concentration (*C*_max_) and area under the plasma concentration-time curve from zero to time *t* (AUC*_t_*) increased dose-dependently; however, log-transformed regression analysis showed that only AUC*_t_* increased dose-proportionally. Tolvaptan *C*_max_ was achieved within 2–3 hours post-dose in each escalating dose group. The half-life (*t*_1/2_) of tolvaptan also increased with each escalating dose group. At the 120 mg dose, mean *t*_1/2_ (11.4 hours) was approximately three-times longer than the mean *t*_1/2_ at the 15 mg dose (3.3 hours). The cumulative urinary excretion rate for tolvaptan also increased up until 48-hour post-dose, though only 0.12%–0.25% of the dose was excreted in the urine. Therefore in a clinical setting, if a patient is dosed with a 15 or 30 mg tablet then peak tolvaptan concentrations will be seen within 2 to 3 hours, with the drug being completely cleared from the body within a 24 hours interval post-dose. In the two multiple-dose, placebo-controlled, parallel-group studies at 30 and 60 mg (double-blind), and at 90 and 120 mg (single-blind), subjects were randomized to receive either one of the two doses of tolvaptan (*n* = 12 total), or placebo (*n* = 6) on Day 1, followed by a 48-hour wash-out period, then once daily for seven days in the fasted state. Accumulation coefficient of the drug ranged from 0.82 to 0.98 across all dose groups, showing that tolvaptan did not accumulate in the plasma after multiple-dosing. There were also no differences seen in fraction of dose excreted unchanged in the urine associated with multiple-dosing. Overall, findings on PK parameters from these data for Japanese subjects were generally comparable and consistent with the results reported by Shoaf *et al.*, which had primarily Caucasian subjects [14].

Kim *et al.* also reported on the effect of food (Japanese standard meal; total calories ~600 Kcal, 2.5 g of sodium) on PK properties of tolvaptan in a randomized, two-group, two-period, open-label, crossover study. Subjects received a 15 mg dose of tolvaptan in the fasted or fed state in the first period (Day 1), followed by a five-day washout, and then received the second treatment (on Day 7). Average time to *C*_max_ under the fed-state was one hour longer than the fasted-state (3.0 *vs.* 2.0 hours). However, *C*_max_ was increased by 28% under the fed-state and AUC*_t_* was increased by 9% in the fed-state compared to the fasted-state [15].

Another similar study assessed the effect of food on tolvaptan PK/PD in healthy Japanese and Caucasian adult male subjects [16]. This was a parallel-group, 3-period, randomized, cross-over trial in which subjects were randomized to receive 30 mg of tolvaptan in the fasted-state, or following a high-fat, high-calorie meal (total calories ~1000 Kcal, 50% from fat), or a Japanese standard meal. The only difference between races was the exposure of tolvaptan, with the coefficient of variation (% CV) for *C*_max_ being lower in the fasted state (29%), compared to the fed states (40%–55%) in both races. Japanese subjects had a higher AUC_∞_ in the fasted and fed states compared to Caucasian subjects. This difference was explained by the smaller body weight in Japanese subjects compared to the Caucasian subjects (64.2 ± 8.4 kg *vs.* 76.4 ± 6.4 kg, respectively). After adjusting for body weight, mean CL/F or AUC was similar between Japanese and Caucasian subjects. In the fasted state, mean CL/F was 5.36 mL/min/kg for Japanese subjects and 5.14 mL/min/kg for Caucasian subjects, and after a high fat meal, it was 4.64 and 4.65 mL/min/kg, respectively. Thus, a high fat meal produced a 1.15-fold increase in plasma tolvaptan concentrations in both races; however a Japanese standard meal produced a 1.15-fold increase only in Japanese subjects compared to the fasted state. This difference in exposure could not be explained by difference in weight, and may be linked to genetic and environmental differences. It should be noted however, that despite the PK differences between the fed and the fasted state in these two studies, no clinically significant PD differences were noted for these two states. Therefore in a clinical situation tolvaptan can be administered with or without food.

These studies only assessed the PK characteristics of tolvaptan in the oral dosage form. To determine absolute bioavailability of tolvaptan, Shoaf *et al.* compared an intravenous (IV) formulation of tolvaptan to the oral formulation in 14 healthy subjects [17]. PK analyses showed that a single administration of a 30 mg oral tolvaptan tablet yielded a mean absolute bioavailability of 56% (range of 42% to 80%).

Tolvaptan is available only as 15 mg or 30 mg tablets in the Unites States (7.5 mg is available in Japan) [2], which presents a challenging problem for patients who are not candidates for oral administration due to dysphagia or other conditions. Our group conducted a randomized, two-treatment crossover study to compare the relative bioavailability and PK of a tolvaptan 15 mg tablet, administered orally as a whole tablet *versus* crushing the tablet and administering via nasogastric (NG) tube in healthy adult subjects [18]. Twenty-eight healthy, fasted subjects received two 15 mg tablets of tolvaptan each given with 240 mL of water (one tablet swallowed intact, and one tablet crushed and given by NG tube), with a minimum 7-day wash-out period. For each period, plasma tolvaptan concentrations were taken from 15 blood samples over 36 hours. Urine output was collected for 24 hours post-dose during each period. The geometric mean ratios, for *C*_max_, AUC*_t_*, and AUC_∞_ were 88.9% (CI 90% 80.1–98.6), 74.3% (CI 90% 68.1–81.0), and 74.2% (CI 90% 68.1–80.9), respectively. It was concluded that when a 15 mg crushed tablet was administered through a dedicated NG tube, CI 90% for AUCs were not within the accepted bioequivalence range of 80%–125%. Although only a modest decrease was seen in *C*_max_ ratio, an approximate 25% decrease was seen in both AUC*_t_* and AUC_∞_ after tolvaptan administration by NG tube. However, while the trial was not designed nor powered to evaluate PD properties of tolvaptan, no observed difference was seen in aquaretic effects of tolvaptan between the two routes of administration. There was only a 2.8% decrease in 24-hour urine output after NG tube administration (6659 mL) compared to the oral tablet swallowed whole (7042 mL) [18]. Oral and NG tube administration of 15 mg tolvaptan to healthy adult subjects was safe and well tolerated, and appeared to produce aquaresis after administration. Effects of NG tube administration on tolvaptan PK and aquaresis in patients with hyponatremia remain to be evaluated. Despite this, with appropriate clinical monitoring, NG tube administration may be a viable approach to administer tolvaptan in patients who are unable to swallow a whole tablet [18].

## 3. Tolvaptan Pharmacodynamics (PD)

Two key PD endpoints most often used to describe tolvaptan’s aquaretic effects are total urine output (L) and urinary excretion rate (mL/h). Other PD parameters include serum and urine electrolyte concentration, serum osmolality, and plasma renin and AVP concentrations. At the time when tolvaptan was approved in 2009, the published study by Shoaf *et al.* suggested that there is saturation in tolvaptan’s effect in urine output and extended duration of aquaretic effect with higher doses. The study team also postulated that the plasma renin and AVP concentrations were not dose-dependently increased by tolvaptan [2,14].

In the single ascending dose study of 15–120 mg by Kim *et al.*, the mean 24-hour cumulative urine output increased dose-dependently, with the 120 mg dose producing up to 4.6 times more urine output than that of placebo [15]. Similarly, the mean 24-hour water intake also increased with increasing dose. The urinary excretion rates rapidly increased and reached maximal rates within 2 to 4 hours post-dose. The maximum mean urinary excretion rates also increased dose-dependently, with peak excretion rate of 700–800 mL/h at doses ≥60 mg. Aquaresis followed the same pattern as the urinary excretion rate and reached a plateau at doses of ≥60 mg within 2 to 4 hours post-dose. The 24-hour cumulative free water clearance showed a dose-dependent increase and reached a plateau at 60 mg as well. At the highest dose of tolvaptan studied, urinary sodium and potassium excretion were also increased compared to placebo group by 1.6 and 2.3 times, respectively. However, no changes in mean serum potassium concentration were found at any of the doses. As the urine osmolality decreased with increasing tolvaptan dose, the mean serum sodium concentration increased from 1.5 to 8.0 mEq/L, with the maximal change from baseline seen at 6 to 8 hours post-dose. The maximal change in serum osmolality from baseline was also seen at 6 hours post-dose in the tolvaptan group.

The multiple-dose studies in the report by Kim *et al.* showed that when tolvaptan was dosed for seven days (Days 3–9), the 24-hour cumulative urine output was decreased only slightly on Day 9 as compared to Day 1 at all dose levels [15]. There was also no change seen in the urine osmolality between Day 1 and Day 7 with multiple-dosing, showing a low likelihood of developing tolerance to tolvaptan. On the other hand, after multiple-dosing in the 30 mg tolvaptan group, mean plasma AVP concentration on Day 9 was similar to that of a single 30 mg dose on Day 1. However, in the 120 mg tolvaptan group, mean plasma AVP concentration was significantly higher on Day 9 than that of a single 120 mg dose on Day 1, even at 24 hours post-dose. This indicates that tolvaptan’s effect retained potency after multiple-dosing. Serum sodium concentrations remained similar between the placebo and low-dose group, but increased in the high-dose group as compared to baseline at Day 9. Serum potassium concentrations remained similar to baseline in the high-dose and low-dose groups as compared to placebo. The results of this study also indicated that at 10 hours post-dose, the urinary excretion rates were similar between 15 mg of tolvaptan and placebo. Additionally, the plasma tolvaptan concentration at 8 hours post a single 15 mg dose correlated with the minimal effective concentration (30 ng/mL) to exert an aquaretic effect, which may be lost after 10 hours. Tolvaptan’s maximal urinary excretion rate was shown to not increase beyond a plasma tolvaptan concentration of 315 ng/mL, which is correlated to the *C*_max_ at 60 mg. While maximal urinary excretion rate plateauted at doses of at least 60 mg, higher doses are needed for extended duration of tolvaptan’s diuretic effect [15].

In another study that assessed the bioavailability of tolvaptan, it was shown to be effective for at least 16 hours after a 30 mg dose when the plasma concentration stayed above 20 ng/mL. In the IV dose group, 1 mg of IV tolvaptan induced an increase in urine output by 1.6-fold compared to placebo for approximately 4 hours, when the concentration ranged from 18–45 ng/mL [17].

Review of tolvaptan PK/PD properties in detail suggest that increasing dose of the drug promotes an increase in the rate of aquaresis and extends the duration of effect, with the maximum urinary excretion rate plateauing at durations of greater than 8 hours post- a 60 mg dose. Based on the studies, these PK/PD parameters were only relevant when tolvaptan was administered alone. Therefore, this review also explored other studies in which tolvaptan concentrations could have been affected due to drug or food interactions.

## 4. CYP3A4 Inhibition and Induction

Tolvaptan was shown to undergo metabolism by cytochrome (CYP) 3A4/5 isoenzymes from *in vitro* studies [2]. In order to fully elucidate the PK and PD of tolvaptan, drug-drug interactions have also been evaluated in clinical studies that explored tolvaptan’s relationship with the co-administration of grapefruit juice, a potent intestinal CYP3A4 inhibitor, ketoconazole, a systemic CYP3A4 inhibitor, and rifampicin, a CYP3A4 inducer.

Tolvaptan 60 mg and grapefruit juice (240 mL) were administered to 20 healthy subjects in a crossover trial, with plasma tolvaptan concentrations being measured at pre-specified time points post-dose [19]. Tolvaptan *C*_max_ and AUC were increased by approximately 50%–85%, while the clearance was reduced by half when tolvaptan was co-administered with grapefruit juice. Plasma tolvaptan concentration was doubled to 16 hours post-dose with co-administration compared to 8 hours with tolvaptan alone. This increase was due to inhibition of the first pass effect in the upper intestine, thus increasing tolvaptan bioavailability. However, plasma tolvaptan concentrations returned to baseline at 24 hours post-dose. In this study, urine output was not measured. However, it was postulated that at a 60 mg dose, grapefruit juice would only increase the exposure of tolvaptan but not further increase the urinary excretion rate. Thus, a 15 or 30 mg dose of tolvaptan may increase urine output when co-administered with grapefruit juice since tolvaptan concentrations would increase the duration of the effect. With a 60 mg dose, the maximum effective concentration is already reached, thus no greater increase in urine output would be seen past the 12 hours time point with co-administration of grapefruit juice [19].

Two other studies performed by Shoaf *et al.* assessed the role of ketoconazole, a systemic CYP3A4 inhibitor, and rifampicin, a CYP3A4 inducer, on the metabolism and effects of tolvaptan using non-compartmental analysis. In the ketoconazole study, 24 healthy subjects were randomized to receive 30 mg tolvaptan (*n* = 19) or placebo (*n* = 15) on Day 1. Ketoconazole 200 mg was administered in the fasted state on Days 4, 5, and 6 along with tolvaptan or placebo on Day 5. Urine and blood samples were taken periodically in order to assess PK and PD parameters of tolvaptan [20]. Plasma tolvaptan *C*_max_ increased from 174 ng/mL to 606 ng/mL; half-life increased from 6.9 hours to 10.5 hours; AUC*_t_* increased from 1360 ng·h/mL to 7670 ng·h/mL; and AUC_∞_ increased from 1460 ng·h/mL to 7880 ng·h/mL in patients that received concomitant ketoconazole. Clearance of tolvaptan was also decreased from 5.63 mL/min/kg to 0.97 mL/min/kg in response to ketoconazole co-administration. Both tolvaptan *C*_max_ and AUC increased (by 3.5-fold and 5.3-fold, respectively) with co-administration of tolvaptan and ketoconazole in healthy subjects, which also had an incremental effect on the PD of tolvaptan. A 1.3-fold increase in urine output at 24 hours was seen in subjects who received both tolvaptan and ketoconazole (4.4 L) compared to those who did not receive ketoconazole (2.7 L). This increased output was likely related to a longer exposure of tolvaptan, as the 30 mg tablet already promoted maximal increase in urine output for 8 hours post-dose. Moreover, the total amount of tolvaptan excreted unchanged was still <1%. Therefore, the effects of low dose tolvaptan co-administered with ketoconazole would be similar to high dose tolvaptan administered alone [20].

Shoaf *et al.* also evaluated the effect of CYP3A4 induction on tolvaptan with rifampicin [20]. 14 healthy subjects were given a single dose of 240 mg tolvaptan with a 48-hour washout followed by a 7-day regimen of 600 mg rifampicin daily, with 240 mg tolvaptan given again on Day 7. Urine output was measured and plasma samples of tolvaptan and rifampicin were analyzed at pre-specified time points. Tolvaptan *C*_max_ decreased from 1000 ng/mL to 168 ng/mL due to rifampicin co-administration. AUC*_t_* also decreased from 11,600 ng·h/mL to 1470 ng·h/mL due to the presence of rifampicin. These results showed that *C*_max_ and AUC*_t_* of tolvaptan were reduced by about 85% when co-administered with rifampicin. However, *t*_1/2_ and CL/F could not be determined due to the large decrease in plasma tolvaptan concentration following co-administration with rifampicin.

The effects of decreased plasma tolvaptan concentration due to CYP3A4 induction also had an effect on urine output. Total urine output for all subjects for a single 240 mg tolvaptan dose showed a 4-fold increase from placebo in the first 24 hours. From 24 to 48 hours, there was also a two-fold increase in total urine output in subjects dosed with tolvaptan 240 mg *vs.* placebo. Following rifampicin co-administration, total urine output pooled together for all 15 subjects at 24 hours decreased from 12,335 mL to 8790 mL, and it was similar to subjects given placebo from 24 to 48 hours. Comparing tolvaptan alone *vs.* tolvaptan plus rifampicin, total urine output was similar at 8 hours post-dose. Rifampicin co-administration, however, decreased mean plasma tolvaptan concentrations at 24 hours to 18 ng/mL, which is lower than the minimal plasma tolvaptan concentration (20–30 ng/mL) needed to produce substantial inhibition of AVP binding to V2 receptors for aquaretic effect [20].

In both trials, no serious or life-threatening adverse events were observed and all treatment-emergent adverse effects (TEAE) were mild to moderate in nature. In the ketoconazole trial, 8 of 19 subjects reported TEAEs in the ketoconazole-tolvaptan co-administration group, compared to 3 of 5 subjects in the placebo-ketoconazole group; with only headache and dizziness occurring in more than one subject. In the rifampicin trial, only nausea and headache were experienced in more than one subject.

These trials indicate that plasma tolvaptan concentration is affected by strong CYP3A4 inhibition and induction. Tolvaptan was shown to produce a maximal effect on urine excretion rate at concentrations >100 ng/mL and appeared to have no aquaretic effect at concentrations <20 ng/mL. Tolvaptan also had a rapid onset and offset of action. Changes in plasma tolvaptan concentration due to drug interactions via CYP3A4 metabolism affected tolvaptan’s half-life and effect on urinary excretion rate. Tolvaptan half-life was increased with ketoconazole co-administration and decreased with rifampicin. Therefore, patients receiving tolvaptan and concurrent CYP3A4 inhibitors or inducers should be monitored closely for increased adverse effects and efficacy of altered aquaretic effect.

## 5. Digoxin and Tolvaptan

Often, patients who require tolvaptan have concomitant cardiovascular medications such as digoxin, warfarin, and loop diuretics that carry significant drug-interaction profiles. In the Phase 3 study, Efficacy of Vasopressin Antagonism in Heart Failure Outcome Study with Tolvaptan: the EVEREST Outcome Trial, 40% of the patients were receiving digoxin at baseline [21]. To better understand the PK of tolvaptan in subjects taking digoxin, Shoaf *et al.* studied the PK interaction between digoxin and tolvaptan [22]. In this study, 14 healthy subjects were administered tolvaptan 60 mg on Day 1, followed by baseline blood and urine analysis. Digoxin was administered with a loading dose of 0.5 mg followed by 0.25 mg daily from Day 5 through Day 15. Tolvaptan was restarted on Day 12 and co-administered along with digoxin through Day 15, and administered alone on Day 16. Blood and urine samples were analyzed at post-dose time points in order to assess PK properties of digoxin and aquaretic effect of tolvaptan.

The study results indicated that the steady state trough digoxin concentration increased from 0.62 ng/mL to 0.75 ng/mL in the presence of tolvaptan, which is within the range of acceptable digoxin trough concentration for therapeutic drug monitoring. The maximum steady state concentration (*C*_ss,max_) and AUC of digoxin were also increased from 1.80 ng/mL to 2.34 ng/mL and 19.9 ng·h/mL to 23.4 ng·h/mL, respectively. Tolvaptan co-administration also decreased digoxin renal clearance from 1.05 mL/min/kg to 0.43 mL/min/kg. Subjects in the study did not suffer from any serious or severe adverse events with the only TEAEs being mild nausea, headache and a non-specific rash.

In order to further elucidate the mechanism of action of tolvaptan on increasing digoxin concentration, *in vitro* studies were conducted in MDR1-expressing LLC-PK1 cells. Digoxin and tolvaptan were co-incubated with MDR1-expressing cell or control cells. Transport studies were conducted to assess the flux ratio between the apical and basal compartments. Verapamil was used as a control to measure the effect of *P*-glycoprotein inhibition on digoxin basal to apical flux ratio. The results of the *in vitro* studies indicated that verapamil 30 µM almost completely inhibited the basal to apical flux of digoxin with a flux ratio of 1.5. Tolvaptan 50 µM also inhibited the basal to apical flux of digoxin with a flux ratio of 1.3. The IC_50_ value for this inhibition was 15.9 µM. These *in vitro* studies indicated that tolvaptan is also a *P*-glycoprotein substrate and inhibits the secretion of digoxin into the proximal convoluted tubule, proportionally decreasing the renal clearance of digoxin. As the study was powered to primarily examine the effect of tolvaptan on digoxin concentration, conclusions could not be drawn about the effects of digoxin on tolvaptan PK properties [22].

Tolvaptan produced a statistically significant, but not clinically significant, increase in digoxin concentration primarily by decreasing the concentration of digoxin available for renal clearance. At steady state, digoxin trough concentrations increased by approximately 20% and peak concentrations increased by 30%. Renal clearance of digoxin was also decreased by 59% in patients due to *P*-glycoprotein inhibition by tolvaptan. It was hypothesized by the study authors that increased 24-hour urine output following concomitant tolvaptan dosing with digoxin might have resulted in more dilute urine samples. This phenomenon could have produced an underestimation of digoxin concentration in the urine and overestimation in reduction of renal clearance of digoxin. However, due to the short duration of tested stability of digoxin in urine, further investigation to validate this phenomenon was not feasible. Therefore even with the neutral safety results obtained from this study, patients receiving tolvaptan and digoxin concomitantly should be monitored for adverse effects related to digoxin [22].

## 6. Warfarin and Tolvaptan

Warfarin is another common concomitant medication in patients, particularly patients with heart failure. Warfarin is mainly metabolized by CYP2C9, CYP2C19, and CYP3A4 isoenzymes. Since tolvaptan is also metabolized primarily by CYP3A4, a study was conducted to evaluate the effect of tolvaptan on warfarin PK/PD. In an open-label, randomized, placebo-controlled crossover study conducted in 23 healthy males and females, subjects received warfarin 25 mg on Day 1, with baseline blood sampling conducted in the clinic in order to assess warfarin PD [23]. Subjects were then administered either tolvaptan 60 mg or placebo once daily from Days 20 through 32 along with warfarin 25 mg on Day 23. Subjects then completed the crossover on Days 34 to 46. Plasma samples were analyzed for determination of Prothrombin Time (PT), Activated Partial Thromboplastin Time (aPTT), and International Normalized Ratio (INR). The results of the study indicated that tolvaptan administration had no statistically significant effect on the concentration-time profile of warfarin. The PD parameters of INR, aPTT, and PT were similar when warfarin was administered with or without tolvaptan. There was also no significant difference in the mean free fraction excretion of tolvaptan before warfarin dosing *vs.* after warfarin dosing (0.77% ± 0.28% *vs.* 0.79% ± 0.14%, respectively) [23]. These results indicated that tolvaptan concentrations were also unaffected by warfarin dosing. All adverse events in the study were mild to moderate in nature with increased urinary frequency and thirst being the most frequent TEAEs. In this study, it was concluded that tolvaptan did not affect warfarin concentration or its relative efficacy. However, additional research in a more clinically relevant setting is necessary to determine the true interaction potential between tolvaptan and warfarin. At this time, close monitoring of PT/INR, efficacy of tolvaptan and other adverse effects should be closely monitored in patients undergoing tolvaptan and warfarin co-administration therapy.

## 7. Diuretics and Tolvaptan

In the EVEREST trial, tolvaptan was evaluated for treatment of clinical congestion in patients with acute heart failure syndromes in addition to standard therapy with loop diuretics [21]. Even though tolvaptan did not show benefits for long-term mortality or heart failure morbidity, the PK/PD parameters of tolvaptan and the conventional treatment of congestion with diuretics in acute heart failure warranted evaluation. In an investigative, 3-period crossover, pilot study, an 80 mg dose of furosemide, a 100 mg of hydrochlorothiazide, and a 30 mg of tolvaptan were administered to 12 subjects to evaluate the PK/PD interactions between tolvaptan and diuretics [24]. Six subjects in Arm 1 all received 30 mg of tolvaptan, 80 mg of furosemide and a combination of tolvaptan (30 mg) plus furosemide (80 mg), with each doses being administered after a 48 hours washout period. Six subjects in Arm 2 were dosed similarly, but with 100 mg hydrochlorothiazide.

In the first part of the study, tolvaptan was dosed alone and in conjunction with furosemide in healthy subjects. Blood and urine samples were collected at pre-specified time points. Bioanalytical analyses were conducted for serum sodium and potassium, plasma rennin activity, and AVP at baseline and at regular intervals thereafter. In the presence of furosemide, tolvaptan *C*_max_ increased by 18% (from 277 ng/mL to 327 ng/mL), and AUC_∞_ increased by 23.5% (from 1.69 µg h/mL to 2.08 µg h/mL). All other PK properties of tolvaptan remained unchanged. Furosemide PK properties also remained unchanged when administered alone or in combination with tolvaptan.

The PD aspects of tolvaptan alone and in combination with furosemide were also analyzed in this study. The cumulative 24-hour urine output for tolvaptan was greater than furosemide alone (4500 mL *vs.* 3200 mL, respectively). When tolvaptan was co-administered with furosemide, the cumulative 24-hour urine output was similar to tolvaptan alone, however the urinary excretion rate and duration of effect for each drug was different when administered alone and in combination. Furosemide peak urinary excretion rate (900 mL/h) was greater than tolvaptan’s peak urinary excretion rate (550 mL/h) for the first 2 hours post-dose, and then decreased to below baseline levels from 6 to 12 hours post-dose. This effect can be attributed to the rebound effect typically seen in studies related to loop diuretics. Conversely, tolvaptan was attributed with a longer duration of effect (12 hours post-dose) compared to furosemide (4 hours post-dose). The co-administration of both drugs increased the urinary excretion rate above baseline from 0 to 12 hours, with the peak excretion rate being equivalent to furosemide’s peak excretion rate. However the urinary excretion rate of co-administered tolvaptan and furosemide from 4 to 12 hours were below the rate seen with tolvaptan alone [24].

Changes in plasma renin activity and AVP concentrations were determined in healthy subjects following oral administration of tolvaptan, furosemide and the combination. From 2 to 24 hours post-dose, mean plasma renin activity was relatively unchanged for subjects dosed with tolvaptan alone but activity was increased for subjects dosed with furosemide alone. The combination of both drugs, however, resulted in a greater increase in mean plasma renin activity than either drug alone at both 2 hours and 24 hours post-dose. Analysis of mean plasma AVP concentrations at 2 hours and 4 hours post-dose showed that, in subjects who received tolvaptan alone, mean plasma AVP concentrations were significantly increased. Mean plasma AVP concentrations remained relatively unchanged in subjects who received furosemide alone. In subjects receiving the combination treatment, mean plasma AVP concentrations were also greater than tolvaptan alone at 2 hours and 4 hours post-dose.

Urine excretion and plasma concentration of electrolytes were also assessed in subjects receiving tolvaptan and furosemide. Urinary excretion of potassium was shown to be increased in all subjects receiving tolvaptan, furosemide and the combination when compared to baseline; however, the 24-hours plasma potassium concentration remained similar between all three treatment groups. Urinary excretion of sodium at 24 hours was shown to be lower than baseline excretion in subjects treated with tolvaptan and higher in subjects treated with furosemide and the combination treatment. The 24-hour plasma sodium concentration for subjects on furosemide and tolvaptan alone remained similar to baseline plasma concentration; however the combination treatment showed an increase in 24-hour plasma sodium concentration by ~3.5 mEq/L compared to baseline. The mean plasma sodium concentration at 6 hours post-dose was increased to 5 mEq/L for tolvaptan and the combination treatment, and it was decreased compared to baseline plasma sodium concentration for subjects receiving furosemide alone [24].

The last aspect of the study was the investigation of free water clearance as seen after administration of tolvaptan or furosemide. For subjects receiving tolvaptan or the combination treatment, free water clearance was increased between 2.2 to 7.0 mL/min at 2 to 4 hours post-dose, indicating that tolvaptan increased free water clearance even in the presence of furosemide. This data also showed that the formation of hypotonic urine was rapid in the presence of tolvaptan. However, furosemide only produced a free water clearance rate of 1.1 mL/min during the same interval.

Safety assessment showed that combined use of tolvaptan and diuretics were well tolerated, with no subjects showing any severe adverse effects related to the medications. TEAEs related to furosemide reported in the study reflect those commonly seen in practice, such as dry mouth, dizziness, dyspepsia, nausea, asthenia, and headache. The only TEAE related to tolvaptan was dyspepsia; and TEAEs related to combination treatment were dizziness and nausea [23]. As this trial was conducted prior to the knowledge of other TEAS related to the mechanism of action of tolvaptan, such as dry mouth, thirst, polyuria and pollakiuria (frequent daytime urination), no data for these adverse events were collected in this early study.

The second part of the study looked to evaluate the PK/PD difference between tolvaptan and a thiazide diuretic. In Arm 2, healthy subjects were dosed with tolvaptan, hydrochlorothiazide and the combination of the two medications, in a 3-period, crossover study [24]. The same PD endpoints were evaluated as in Arm 1. The PK results indicated that no changes in concentration, AUC, half-life and clearance were seen when tolvaptan or hydrochlorothiazide were dosed alone compared to concomitant dosing.

The PD differences between tolvaptan and hydrochlorothiazide also indicated a similar pattern to the results obtained from Arm 1. Cumulative 24-hour urine output for tolvaptan (4000 mL) was also higher than the cumulative 24-hour urine output for hydrochlorothiazide (3500 mL). This increase in urine output for tolvaptan alone was an approximate 50% increase from baseline. Concomitant dosing of tolvaptan and hydrochlorothiazide did not significantly increase the cumulative 24-hour urine output compared to tolvaptan alone. The maximal urinary excretion rate for tolvaptan and hydrochlorothiazide were similar when dosed alone and with concomitant dosing. The duration of effect of both medications was also similar when dosed alone and in combination, indicating a longer duration of effect when compared with a loop diuretic such as furosemide. There was also no indication of a rebound effect seen with the use of a thiazide diuretic as seen with furosemide in Arm 1 of the study [24].

The neurohormonal activation seen in Arm 1 of the study with tolvaptan was also evident in this arm and yielded similar results. Treatment with hydrochlorothiazide yielded similar effects on renin activity as treatment with furosemide. Mean renin activity increased by 4.4 ng/mL/h with hydrochlorothiazide treatment alone, and increased by 5.0 ng/mL/h with combination of hydrochlorothiazide and tolvaptan. Plasma AVP concentration with tolvaptan dosing increased just as in Arm 1 of the study; however, unlike furosemide, hydrochlorothiazide was shown to decrease AVP concentrations compared to baseline at 2 and 4 hours. The combination treatment of hydrochlorothiazide and tolvaptan followed a similar pattern to Arm 1 with an increase in plasma AVP concentration seen at 2 and 4 hours, albeit to a lesser extent compared to concomitant dosing with furosemide.

Urinary excretion of electrolytes and their subsequent plasma concentrations were also assessed as part of this study. As with Arm 1, urinary excretion of potassium was increased for all three treatment groups when compared to baseline, however, no change was seen in the plasma concentration of potassium in subjects receiving these treatments. The excretion of sodium also followed a similar pattern as Arm 1, with urinary excretion of sodium being lower compared to baseline in subjects who received tolvaptan, and the excretion being higher than baseline in subjects who received hydrochlorothiazide and the combination therapy. These results also indicated that in this study, tolvaptan, due to the low amount of urinary sodium excretion, was the only drug that allowed for the formation of hypotonic urine compared to furosemide and hydrochlorothiazide. In Arm 2 of the study the 24-hour plasma sodium concentrations were similar to baseline for tolvaptan and hydrochlorothiazide when dosed alone, and only increased by 2 mEq/L when dosed concomitantly.

The final assessment of the study was the rate of free water clearance after the administration of tolvaptan, hydrochlorothiazide and the combination treatment. As seen in Arm 1, subjects administered tolvaptan had a maximum rate of free water clearance of approximately 4.5 mL/min within 4 hours post-dose. A similar rate was also noticed in patients given tolvaptan and hydrochlorothiazide concomitantly in the same time frame. However, when hydrochlorothiazide was administered alone, free water clearance decreased within 4 hours post-dose [24]. These PK/PD differences in Arm 2 did not produce severe adverse effects in subjects. The common TEAEs seen with tolvaptan were similar to adverse effects identified in other studies, such as dry mouth and thirst. For subjects treated with hydrochlorothiazide, the most common adverse effect was also thirst; and concomitant dosing of both drugs was associated with increased somnolence and dry mouth.

In conclusion, tolvaptan did not adversely affect the PK of either diuretic in healthy subjects. The PD assessments indicated that 30 mg tolvaptan provided greatest 24-hour urine output as compared with either 80 mg furosemide or 100 mg hydrochlorothiazide; and the combination treatment with either diuretic did not provide additional urine output compared to tolvaptan. In this study, tolvaptan also had an increased duration of effect as compared with furosemide, a drug that suffers from a rebound effect; however tolvaptan had a similar duration of effect when compared to hydrochlorothiazide. Endogenous renin activity was increased when subjects were dosed with furosemide as compared to tolvaptan, indicating an activation of renin angiotensin system with the loop diuretic but not with tolvaptan. Plasma potassium and sodium concentrations were also maintained at clinically safe concentrations with the administration of any of the treatment modalities. The co-administration of tolvaptan with either diuretic was also not associated with serious adverse effects, indicating that tolvaptan was safe and well tolerated at the administered dose and when dosed with furosemide or hydrochlorothiazide.

## 8. Summary and Conclusions

Tolvaptan is an orally active non-peptide AVP V2 receptor blocker that is clinically used for the treatment of hypervolemic and euvolemic hyponatremia or less marked hyponatremia that is symptomatic and has resisted correction with fluid restriction [1]. Since the FDA approval in 2009, additional studies have been performed to increase understanding of PK and PD properties of tolvaptan dosed with concomitant drugs and food. These clinical and *in vitro* studies, in conjunction with the original PK/PD studies reported prior to the drug’s approval, have allowed for better understanding of the relationship between tolvaptan’s PK and PD [14]. Review of these studies showed that PD changes are not dose-proportional to PK because of the saturation of aquaretic response. Thus, with proper monitoring, patients can be safely dose escalated from 15 to 60 mg, the maximum effective dose, for multiple days and experience the desired aquaretic effect. Moreover, no major safety concerns have been noted with higher doses. In terms of administration, food was not shown to significantly affect tolvaptan PK; and tolvaptan may be safely administered via NG tube in patients who may not be able to swallow the drug orally [15,18]. Concomitant use of tolvaptan with strong CYP3A4 inhibitors is contraindicated, and concomitant use with moderate CYP3A4 inhibitors should be avoided due to a lack of studies and experience to define a safe dose adjustment [2]. Likewise, co-administration of tolvaptan with CYP3A4 inducers should be avoided whereas co-administration with digoxin or warfarin did not demonstrate significant clinical effects in healthy subjects [22,23]. If co-administered with CYP3A4 inducers, the tolvaptan dose may need to be increased; but clinical studies and experience are also not adequate to define a safe and effective dose adjustment. Tolvaptan increased urine output in patients compared to furosemide or hydrochlorothiazide in a safe and effective manner; and co-administration with these diuretics was safe and well tolerated in patients [24]. Through a thorough review of clinical, PK and PD studies, it can be ascertained that tolvaptan is a safe and effective therapy that can be utilized effectively in a clinical setting to induce aquaresis in patients with hypervolemic and euvolemic hyponatremia.

## References

[1] New Drug Application for Tolvaptan, Otsukas Investigational Novel Oral Treatment for Worsening Heart Failure and Hyponatremia, Accepted by the U.S. Food and Drug Administration. http://www.prnewswire.com/news-releases/new-drug-application-for-tolvaptan-otsukas-investigational-novel-oral-treatment-for-worsening-heart-failure-and-hyponatremia-accepted-by-the-us-food-and-drug-administration-58860972.html.

[2] Rockville M.D. (2009). Samsca® [package insert].

[3] Saito T., Ishikawa S., Abe K. (1997). Acute aquaresis by the nonpeptide arginine vasopressin (AVP) antagonist OPC-31260 improves hyponatremia in patients with syndrome of inappropriate secretion of antidiuretic hormone (SIADH). J. Clin. Endocrinol. Metab..

[4] De Luca L., Klein L., Udelson J.E. (2005). Hyponatremia in patients with heart failure. Am. J. Cardiol..

[5] Sica D.A. (2005). Hyponatremia and heart failure—Pathophysiology and implications. Congest. Heart Fail..

[6] Gheorghiade M., Gattis W.A., O’Connor C.M. (2004). Effects of tolvaptan, a vasopressin antagonist, in patients hospitalized with worsening heart failure: A randomized controlled trial. JAMA.

[7] Wu C.C., Yeung L.K., Tsai W.S. (2006). Incidence and factors predictive of acute renal failure in patients with advanced liver cirrhosis. Clin. Nephrol..

[8] Gerbes A.L., Gulberg V., Gines P. (2003). Therapy of hyponatremia in cirrhosis with a vasopressin receptor antagonist: A randomized double-blind multicenter trial. Gastroenterology.

[9] Gheorghiade M., Gottlieb S.S., Udelson J.E. (2006). Vasopressin V_2_ receptor blockade with tolvaptan *versus* fluid restriction in the treatment of hyponatremia. Am. J. Cardiol..

[10] Gheorghiade M., Niazi I., Ouyang J. (2003). Vasopressin V_2_-receptor blockade with tolvaptan in patients with chronic heart failure: Results from a double-blind, randomized trial. Circulation.

[11] Goldsmith S.R. (2005). Current treatments and novel pharmacologic treatments for hyponatremia in congestive heart failure. Am. J. Cardiol..

[12] Guyton A.C., Hall J.E. (2000). Textbook of Medical Physiolog.

[13] Lee C.R., Watkins M.L., Patterson J.H. (2003). Vasopressin: A new target for the treatment of heart failure. Am. Heart J..

[14] Shoaf S., Wang Z., Bricmont P. (2007). Pharmacokinetics, pharmacodynamics, and safety of tolvaptan, a nonpeptide AVP antagonist, during ascending single-dose studies in healthy subjects. J. Clin. Pharmacol..

[15] Kim S., Hasunuma T., Sato O. (2011). Pharmacokinetic, pharmacodynamic and safety of tolvaptan—A novel, oral, selective non-peptide AVP V_2_-receptor antagonist: Results of single- and multiple-dose studies in healthy Japanese male volunteers. Cardiovasc. Drugs Ther..

[16] Shoaf S., Kim S., Bricmont P. (2012). Pharmacokinetics and pharmacodynamics of single-dose oral tolvaptan in fasted and non-fasted states in healthy Caucasian and Japanese male subjects. Eur. J. Clin. Pharm..

[17] Shoaf S., Bricmont P., Mallikaarjun S. (2012). Absolute bioavailability of tolvaptan and determination of minimally effective concentrations in healthy subjects. Int. J. Clin. Pharmacol. Ther..

[18] McNeely E.B., Talameh J.A., Adams K. (2013). Relative bioavailability of tolvaptan administered via nasogastric tube and tolvaptan tablets swallowed intact. Am. J. Health Syst. Pharm..

[19] Shoaf S., Mallikaarjun S., Bricmont P. (2012). Effect of grapefruit juice on the pharmakokinetics of tolvaptan, a non-peptide arginine vasopressin antagonist, in healthy subjects. Eur. J. Clin. Pharmacol..

[20] Shoaf S., Bricmont P., Mallikaarjun S. (2012). Effects of CYP3A4 inhibition and induction on the pharmacokinetics and pharmacodynamics of tolvaptan, a non-peptide AVP antagonist, in healthy subjects. Br. J. Clin. Pharmacol..

[21] Konstam M.A., Gheorghiade M., Burnett J.C. (2007). Effects of oral tolvaptan in patients hospitalized for worsening heart failure: EVEREST outcome trial. JAMA.

[22] Shoaf S., Ohzone Y., Ninomiya S. (2011). *In vitro P*-glycoprotein interaction and steady-state pharmacokinetic interactions between tolvaptan and digoxin in healthy subjects. J. Clin. Pharmacol..

[23] Shoaf S., Mallikaarjun S. (2012). Pharmacokinetic and pharmacodynamic interaction between tolvaptan and warfarin in healthy subjects. Clin. Pharmacol. Drug Dev..

[24] Shoaf S., Bramer S., Bricmont P. (2007). Pharmacokinetic and pharmacodynamics interaction between tolvaptan, a non-peptide AVP antagonist, and furosemide or hydrochlorothiazide. J. Cardiovasc. Pharmacol..

